# The History of Mankind

**Published:** 1900-03

**Authors:** 


### Reviews of Books.

**The History of Mankind.** By Professor FRIEDRICH RATZEL. Translated from the Second German Edition by A. J. BUTLER. With Introduction by E. B. TYLOR, D.C.L., F.R.S. Three volumes: xxiv., 486; xiv., 562; xiii., 599. London: Macmillan and Co., Ltd. 1896–1898.

Professor Ratzel's work is one of those encyclopædic productions in the domain of anthropology of which our own fellow-citizen James Cowles Prichard gave us the first example, followed by Waitz, J. G. Wood, and Robert Brown. The present work was well received in Germany on its first appearance more than ten years ago, and was well worthy of the translation which has been executed from the revised edition published in 1894-6.

Physical anthropology was later in its inception as a study than ethnography; but for a time it surpassed the latter in its progress and in the interest taken in it by the leaders of the science. But of late years this has ceased to be the case, especially in England, where, notwithstanding the wide field offered by our extensive colonies and foreign possessions, comparatively little work is now being done in physical anthropology. Indeed the Russians, the Italians, and even the Spaniards, have latterly done more and better work in this part of the field than the English or the Germans. On the other hand the study of the arts and customs of mankind, primeval or contemporary, of the æsthetic, archæological and social departments of the subject, has been prosecuted with ever-increasing vigour and success. As Dr. Tylor, in his interesting and only too short introduction, puts it, "in our time there has come to the front a special study of human life through such object-lessons as are furnished by the specimens in museums," things which used to be looked on as little more than curiosities and hardly suspected of being instructive.

Professor Ratzel's programme is "to impart a knowledge of mankind as we find it to-day throughout the earth," and more especially a knowledge of the "lower strata of humanity;" and "to trace actually among these lower strata the processes which have rendered possible the transition to the higher developments [in the superior races] of to-day. Ethnography must acquaint us not only with what man is, but with the means by which he has become what he is, so far as the process has left any traces of its manifold inner workings." Hereupon he makes the very true and pregnant, but by no means superficial, observation that "the difference of civilization which divides two groups of mankind may bear no kind of relation to the difference of their endowments." He goes further, and after mentioning Nachtigal's Tedas of Fezzan, the Garamantian Troglodytes of Herodotus, who are at this day in the same stage wherein Herodotus knew of them, and after contrasting their history with that of our own kindred, he puts this momentous question: "Are we [as individuals], in physical or intellectual power, in virtue, in capacity, any further ahead of our generations of ancestors than the Tubus [or Tedas] of theirs?" Evidently he thinks we are not; and the followers of Weissman ought to agree with him.

To general considerations of this kind scarcely a tenth part of these three good-sized volumes is dedicated. We have not space to spare for Professor Ratzel's generalisations, which indeed are given so succinctly, as a rule, that they are hardly capable of condensation. His opinions, often original, sometimes, as we think, erroneous, but never baseless, are very clearly expressed. Thus, "In the high plateaux of Mexico and Upper Peru we have land less fruitful than the surrounding lowlands; and accordingly on these plateaux we find the highest development of civilisation in all America." "Another mode in which civilisation may perish is through the absorption of higher races by lower, who profit by the advantage of better adaptation to conditions of hardship." "In North America rapid decay befell all native industrial activity, as the result of the introduction by white men of more suitable tools, vessels, clothing and so forth." "Every people can learn the language of every other; and therein the civilised races have no absolute superiority over the savage." "The highest cultivation of gesture-language seems to be reserved for the inventive, but at the same time, taciturn Indians of North America." "Let us remember how in Abyssinian Christianity, Mongolian Buddhism, Sudanese Mahommedanism, great religious thoughts have dwindled away beyond recognition. The propagative force of religious ideas is as great as the certainty that they will dwindle when they are cast forth into the wilderness of the materialistic savage life." "The primitive temple more often grew out of the churchyard than had the churchyard appended to it."

The above are specimens of Professor Ratzel's opinions. But by far the greater part of his work is not made up, or even largely interspersed with, such generalities: it consists of masses of details respecting the physical characteristics, manners and customs, dress, weapons, houses, religious views and observances of the different tribes, nations or races. Like Topinard, he is fond of laying stress on the impossibility of absolutely pure races: he sees everywhere the outcome of ancient or modern mixtures and crosses. In following out this idea he gives an interesting account of the migrations of the Polynesians and Micronesians, and puts forward conjectures as to the origin and composition of the Australian people, and as to the migrations, counter-migrations, amalgamations and cleavages of the innumerable tribes of Africa. On those of the nomads of Central Asia he is less explicit: the subject is so difficult as to be somewhat repulsive. He quotes, however, an interesting and significant Tibetan legend. A man roamed over the steppes with his three sons. When he died, each one of the three wanted to have the body, in order to bury it in his own fashion: "this was the first quarrel. The eldest got the head, went eastward, and became the father of the cunning Chinese. The second was content with the limbs [the locomotive apparatus] of the deceased. He left his home and settled where the enormous deserts allowed his posterity, the Mongols, room enough to move about. The youngest took the breast and stomach" (and, we suppose, the heart and liver, the last being the supposed seat of the affections); "and from him are descended the people of Tibet, who in daily intercourse are distinguished by good nature, frankness, and cordial feeling, and in war by valour and courage."

With the physical side of anthropology Professor Ratzel is not so much concerned as with some other departments; but any deficiency on that score is made up for by the very numerous and excellent reproductions of photographic portraits, many of which, said to be from the collection of Pruner Bey, we recognise as the handiwork of that modest and industrious labourer in the field, the late M. Potteau of the Jardin des Plantes. As regards complexion, Professor Ratzel seems to attribute intellectual superiority and masterly qualities almost invariably to the fairer races and types, though the darker ones are sometimes superior in the industrial arts.

We cannot dismiss this excellent book without another word of appreciation for the numerous and beautiful illustrations, especially the coloured representations of furniture, weapons, domestic implements, &c., printed by the Bibliographisches Institut, Leipzig. The translator also well deserves his share of praise.

